# Effectiveness of a BCW theory-based exercise adherence intervention protocol in Chinese maintenance hemodialysis patients: a randomized controlled trial

**DOI:** 10.3389/fpubh.2025.1700057

**Published:** 2025-12-12

**Authors:** Qianqian Wei, Hailin Zhang, Fei Chen, Yanan Ban, Xiaoyan Wen, Lixia Yin

**Affiliations:** 1 Blood Purification Centre, Lianyungang Clinical College of Nanjing Medical University, Lianyungang, China; 2 Nursing Department, The Fourth People’s Hospital of Lianyungang, Lianyungang, China; 3 Blood Purification Centre, The Affiliated Lianyungang Hospital of Xuzhou Medical University, Lianyungang, China

**Keywords:** maintenance hemodialysis, behavior change wheel, exercise, adherence, mHealth technology

## Abstract

**Objective:**

To develop and implement a theory-based exercise adherence intervention grounded in the behavior change wheel (BCW) framework, with the goal of improving exercise adherence among maintenance hemodialysis (MHD) patients, enhancing clinical outcomes, and maximizing the benefits of physical activity.

**Methods:**

This study included two phases. In Phase I (Development), we used the BCW framework to develop the intervention. This process involved semi-structured patient interviews and evidence synthesis, followed by refinement with an expert panel. In Phase II (Evaluation), we performed an assessor-blinded randomized controlled trial (RCT). We randomly assigned 80 MHD patients (1:1) to either the intervention or control group. A computer-generated sequence determined allocation, which was concealed with opaque, sealed envelopes. The intervention group received the BCW-based exercise protocol via mobile health (mHealth) technology, and the control group received standard nursing care. Throughout the trial, outcome assessors and data analysts remained blinded to group assignment.

**Results:**

Following the 12-week intervention, the intervention group demonstrated significantly greater exercise adherence scores compared to controls (*p* < 0.001). Key outcome measures demonstrated statistically significant improvements in the intervention group, including: increased average daily step count (*p* < 0.001), enhanced grip strength (*p* = 0.004), improved 6-meter walking speed (*p* < 0.001), greater 6-min walk distance (*p* < 0.001), higher skeletal muscle mass index (*p* = 0.019), improved dialysis adequacy (Kt/V, *p* = 0.034), and elevated serum albumin levels (*p* = 0.009). The intervention group also showed significantly lower triglyceride-glucose (TyG) index values (*p* = 0.004). No significant changes were observed in inflammatory markers (*p* > 0.05).

**Conclusion:**

The study findings demonstrate the efficacy of a BCW-based exercise adherence intervention program in enhancing exercise adherence, physical functioning, dialysis adequacy, nutritional status, and glucose-lipid metabolism among MHD patients.

**Clinical trial registration:**

https://www.chictr.org.cn; identifier (ChiCTR2400091015).

## 1 Introduction

With advancing medical technologies, the population of maintenance hemodialysis (MHD) patients has grown substantially while achieving improved survival rates (1). However, this clinical success has been accompanied by significant challenges, including progressive physical functional decline and complications such as sarcopenia, malnutrition, and cognitive frailty (2–4). All these factors substantially impair patients’ quality of life and physical and psychological well-being, while imposing a heavy burden on public health systems and socioeconomic resources. Exercise serves as a cornerstone of renal rehabilitation for patients on MHD. By improving muscle strength, enhancing dialysis efficacy, and addressing nutritional status, it helps delay the onset of complications and plays a vital role in disease prognosis (5–8). Consequently, clinical practice guidelines across many countries recommend that eligible MHD patients engage in physical activity when tolerated and deemed safe (9–11).

However, the positive physiological effects of exercise are entirely contingent upon patients’ long-term adherence to exercise regimens. Despite the potential benefits for MHD patients, overall physical activity levels remain suboptimal, with low adherence rates reported (12). Research data indicate that over 40% of patients never engage in exercise (13, 14), nearly half maintain low activity levels (15), and only 6% sustain regular weekly exercise (16). This trend is particularly evident within the Chinese MHD population, characterized by a significant disparity between high exercise intention and low participation rates, coupled with concerningly high dropout rates (17–19). Furthermore, exercise rehabilitation practices in MHD patients lag notably behind those observed in other chronic disease populations (e.g., diabetes, hypertension, stroke) (20). Evidence indicates that exercise adherence in MHD patients is influenced by multidimensional factors, encompassing fatigue and diminished muscle strength at the physiological level, depression and anxiety as psychological barriers, and deficiencies in environmental and social support systems (9, 21). Physical inactivity initiates a cascade of adverse effects in MHD patients, accelerating muscle loss, diminishing physical function, and reducing exercise capacity. This decline subsequently undermines exercise adherence and further compromises functional status, establishing a self-perpetuating cycle that ultimately increases disease severity and mortality risk (22).

To address this challenge, various strategies have been explored. For instance, the implementation of a Comprehensive Exercise (COMEX) Intervention (23) has demonstrated the potential of structured programs in improving adherence. Similarly, the use of wearable devices for activity monitoring combined with structured feedback has been shown to effectively enhance patients’ daily activity levels (24). However, these studies are limited by small sample sizes, lack of personalized prescriptions, and insufficient grounding in behavioral theory. Moreover, they have not adequately addressed key psychological constructs such as self-efficacy within the behavior change process. Although theoretical models including the Knowledge-Attitude-Practice model (25), Self-Determination Theory (26), and the ADOPT model (27) have been applied in some Chinese studies, their interventional focus remains predominantly on individual-level cognitive modification or motivational enhancement. These approaches fail to systematically integrate the broader social support networks and external environmental factors that influence patient behavior, thereby limiting the sustainability of intervention effects.

**Figure 1:**
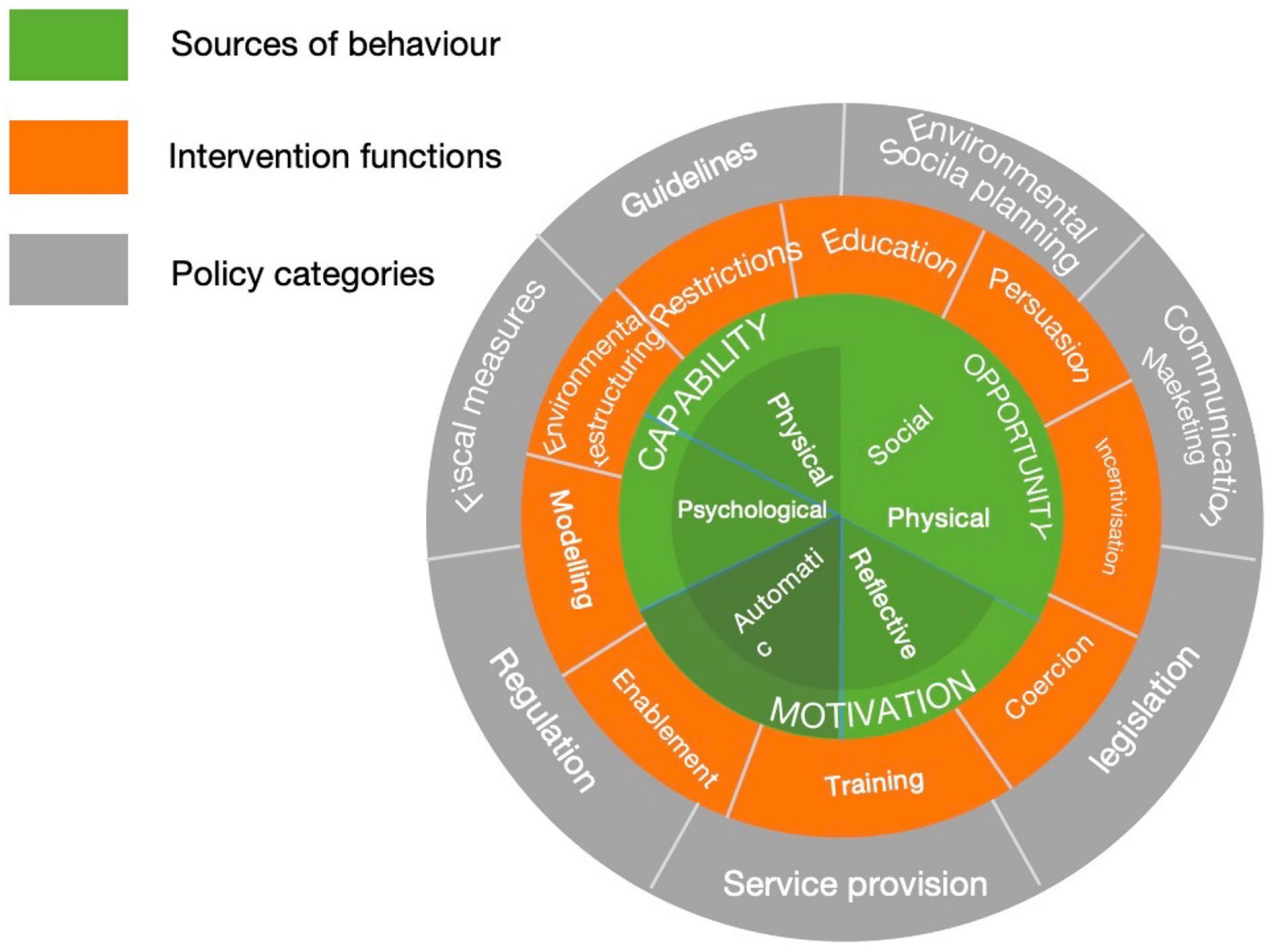
The behavior change wheel framework (adapted from Michie et al. (28)).

**Figure 2:**
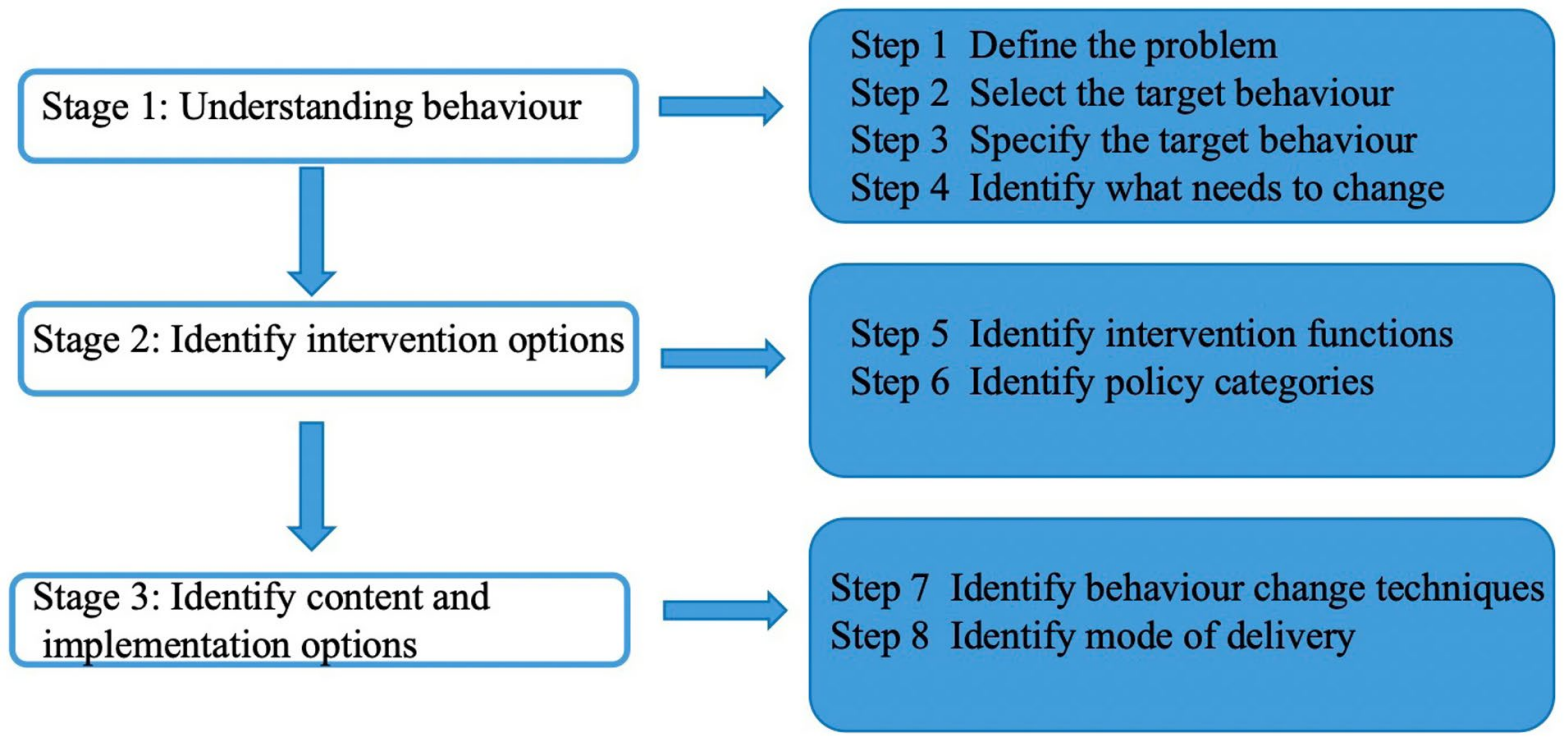
BCW-based intervention development process.

The behavior change wheel (BCW) (28) offers a synthesized framework derived from 19 established behavior change theories. It is structured in three layers (Figure 1): the core consists of the COM-B model (Capability, Opportunity, Motivation–Behavior); the middle layer includes nine intervention functions (e.g., education, incentivization, training) along with 93 behavior change techniques (BCTs), which support behavior change through targeted strategies; and the outer layer comprises seven policy categories (e.g., guidelines, fiscal measures, legislation) (29) that facilitate the implementation of interventions. Guided by this model, the three main implementation steps (Figure 2) provide a comprehensive and systematic framework for designing behavioral interventions, improving both their feasibility and standardization.

The BCW has demonstrated significant efficacy in health behavior promotion and chronic disease management. Evidence indicates that this framework effectively enhances treatment adherence and rehabilitation outcomes in populations such as individuals with diabetes and orthopedic postoperative patients (30–32), while also showing positive results in self-management domains (33–36). Although the BCW has been successfully applied to fluid management and self-care among MHD patients (37, 38), its potential for improving exercise adherence in this population remains underexplored.

Therefore, this study aims to develop and evaluate a BCW theory-based intervention protocol to improve exercise adherence in MHD patients. The research will be conducted in two phases: Phase I will systematically develop the intervention through semi-structured interviews, systematic evidence synthesis, and expert consensus panels; Phase II will empirically test the protocol’s effectiveness through a randomized controlled trial, with comprehensive evaluation across multiple domains including exercise adherence, physical function, body composition, and laboratory biomarkers. The findings will provide both theoretical foundation and practical guidance for enhancing exercise adherence in this clinical population.

## 2 Methods

### 2.1 Phase I: development of the exercise adherence intervention protocol

Guided by the BCW framework, this phase systematically constructed the intervention protocol through three sequential steps: (1) identifying core determinants and establishing an initial intervention framework via semi-structured interviews; (2) refining specific intervention components based on a synthesis of best available evidence; and (3) finalizing the protocol through structured expert consensus meetings.

#### 2.1.1 Semi-structured interviews implementation

Guided by the COM-B model, this phase employed semi-structured interviews to explore factors influencing exercise engagement willingness and management needs among MHD patients, thereby informing the development of a targeted intervention protocol. The study received ethical approval from the Institutional Review Board (approval no: KY-20240409001-02).

The interview guide, developed through literature review and structured by the COM-B model, included the following domains: (1) “Are you aware of exercise rehabilitation? What types of exercise are suitable for you?” (2) “Do you consider your current physical condition appropriate for exercise? What benefits do you associate with exercise?” (3) “Do you currently exercise? If so, what facilitates your engagement? If not, what barriers exist?” (4) “What exercise modalities do you prefer? Do you receive support from family, friends, or healthcare providers?” (5) “Would you participate in a dialysis center-based exercise program?” (6) “What support would you need to engage in regular exercise?”

Using purposive sampling, we conducted semi-structured interviews with 13 eligible MHD patients between April and May 2024. Prior to each interview, participants received a detailed explanation of the study purpose and provided written informed consent. Participants were sequentially coded as P1 to P13 based on interview order. Two researchers independently transcribed the audio-recorded interviews verbatim and analyzed the data using Colaizzi’s seven-step phenomenological method (39) for thematic extraction. The resulting themes, structured within the COM-B model, formed an initial intervention framework that directly guided the subsequent choice of BCW intervention functions.

#### 2.1.2 Summary of best evidence

This phase aimed to systematically retrieve and evaluate existing evidence on improving exercise adherence in MHD patients, thereby providing a practical foundation for program development. This study was prospectively registered at the Fudan University Center for Evidence-Based Nursing (Registration No. ES20244851). The process, detailed in Supplementary, involved three sequential steps:

##### 2.1.2.1 Question formulation and search

The evidence-based question was structured using the PIPOST framework (40), which articulates the key components: Population, Intervention, Professionals, Outcomes, Setting, and Timeframe. This framework was used to systematically define all components of the clinical scenario, ensuring the retrieved evidence would be directly applicable to the target population, professionals, and settings (Supplementary Table S1). A comprehensive literature search was conducted from database inception to July 2024, guided by the 6S evidence pyramid model. This model, which encompasses six sequential layers (Systems, Summaries, Synopses of Syntheses, Syntheses, Synopses of Studies, and Studies), was applied by adopting a top-down search strategy. This approach ensures the initial identification of the most synthesized and authoritative evidence, thereby optimizing the quality of our evidence base.

##### 2.1.2.2 Quality appraisal

The methodological rigor of the included evidence was critically evaluated using internationally recognized tools. Clinical practice guidelines were appraised using the AGREE II instrument (41), which comprises 23 items across 6 domains. Each item is rated on a 7-point scale (1 = Strongly Disagree to 7 = Strongly Agree). Domain scores are calculated as standardized percentages, and guidelines are assigned a recommendation grade as follows: Grade A (all domain scores >60%), Grade B (**≥**3 domain scores between 30 and 60%), and Grade C (**≥**3 domain scores <30%). To uphold the methodological rigor of this study, guidelines with a Grade C recommendation were considered low quality and were excluded from the evidence base. For systematic reviews, expert consensuses, and randomized controlled trials, the corresponding Joanna Briggs Institute (JBI) critical appraisal tools (42) were employed. These tools utilize design-specific checklists where each item is judged as “Yes,” “No,” “Unclear,” or “Not Applicable,” ensuring a consistent and rigorous evaluation. Two researchers independently performed the literature quality assessment, with disagreements resolved by consensus or, when necessary, by a third researcher who made the final decision.

##### 2.1.2.3 Evidence grading and selection

The level of evidence (1–5) for each study was initially assigned using the Joanna Briggs Institute (JBI) evidence pre-classification system (43), which categorizes study designs as follows: Level 1 (randomized controlled trials or experimental studies), Level 2 (quasi-experimental studies), Level 3 (analytical observational studies), Level 4 (descriptive studies), and Level 5 (expert opinion or basic research). Subsequently, the final evidence selection was determined through a formal expert consensus process, during which the JBI Strength of Recommendation classification (Grade A or B) was applied. The panel comprised six members (a nephrologist, a cardiologist, a rehabilitation technologist, and two hemodialysis nurses), including two PhDs, three Masters, and one Bachelor’s degree holder, all possessing over 10 years of experience in their respective fields. This composition ensured the representativeness and reliability of the consensus process. The expert panel evaluated each piece of evidence in terms of its feasibility, appropriateness, meaning, and effectiveness. Only evidence that received a Grade A (strong recommendation) or B (weak recommendation) was included in the final synthesis.

The selected evidence was synthesized and used to refine and substantiate the intervention framework derived from the qualitative interviews, forming the preliminary draft of the protocol.

#### 2.1.3 Expert panel consensus

To refine the preliminary protocol and enhance its clinical applicability, we convened a structured expert panel to establish consensus on its feasibility, safety, and implementation.

Expert panelists were selected based on the following predetermined criteria: (1) bachelor’s degree or higher with intermediate professional title or above; (2) minimum 5 years of clinical experience in nephrology, exercise rehabilitation, cardiovascular disease, psychology, or nursing; (3) voluntary participation. The final panel comprised 14 multidisciplinary experts: three nephrologists, two exercise therapists, two cardiologists, one psychologist, two nursing administrators, and four hemodialysis nurses.

One week prior to the scheduled meeting, we distributed the draft intervention protocol and structured evaluation forms via email for independent review. The meeting agenda focused on three key domains: (1) overall protocol rationale and coherence, (2) scientific validity and operational practicality of specific components, and (3) additional suggestions for improvement. Following the meeting, all feedback from the discussion transcripts and expert evaluation forms was systematically collated and analyzed. The protocol underwent iterative revision based on this synthesized expert input, resulting in the finalized version.

Expert engagement was quantified by the questionnaire return rate. The authority coefficient (Cr) was calculated as the arithmetic mean of an expert’s familiarity with the content (Cs) and their basis for judgment (Ca). The degree of consensus among panelists was measured using Kendall’s coefficient of concordance (W).

In summary, the Phase I systematically developed the “Exercise Adherence Intervention Protocol for MHD Patients” through the three described steps. The resulting comprehensive protocol provides the foundational intervention for Phase II, which will proceed to empirically evaluate its effectiveness in improving exercise adherence and clinical outcomes in a randomized controlled trial.

### 2.2 Phase II: application of the exercise adherence intervention protocol

#### 2.2.1 Eligibility criteria

##### 2.2.1.1 Inclusion criteria

(1) Aged **≥**18 years; (2) Received regular hemodialysis for >3 months and maintained a stable clinical status during the 4 weeks preceding enrollment, defined as follows: Pre-dialysis systolic blood pressure maintained between 90 and 160 mmHg, with interdialytic weight gain controlled within 5% of dry weight; Absence of acute complications: no occurrence of symptomatic hypotension, acute heart failure, unstable angina, or other acute clinical events; Treatment regimen stability: no requirement for adjustments to dialysis prescriptions or antihypertensive medications due to acute clinical deterioration; (3) No musculoskeletal disorders, ability to ambulate independently; (4) Patient or primary caregiver able to communicate effectively; (5) Smartphone proficiency (patient or caregiver).

##### 2.2.1.2 Exclusion criteria

(1) Uncontrolled hypertension (>180/100 mmHg), hypotension (<90/60 mmHg), or pulmonary hypertension; (2) Acute infections, communicable diseases, malignancies, or other critical illnesses; (3) Concurrent angina pectoris, atrial fibrillation, tachycardia, or other severe arrhythmias, as well as frequent intradialytic hypotension; (4) Symptoms of deep vein thrombosis (DVT); (5) Severe edema (defined as bilateral pitting edema extending to or above the level of the knee); osteoarticular diseases; or fractures within the past 6 months that impair limb function; (6) Presence of metallic implants or electronic medical devices.

#### 2.2.2 Participant recruitment

**Figure 3:**
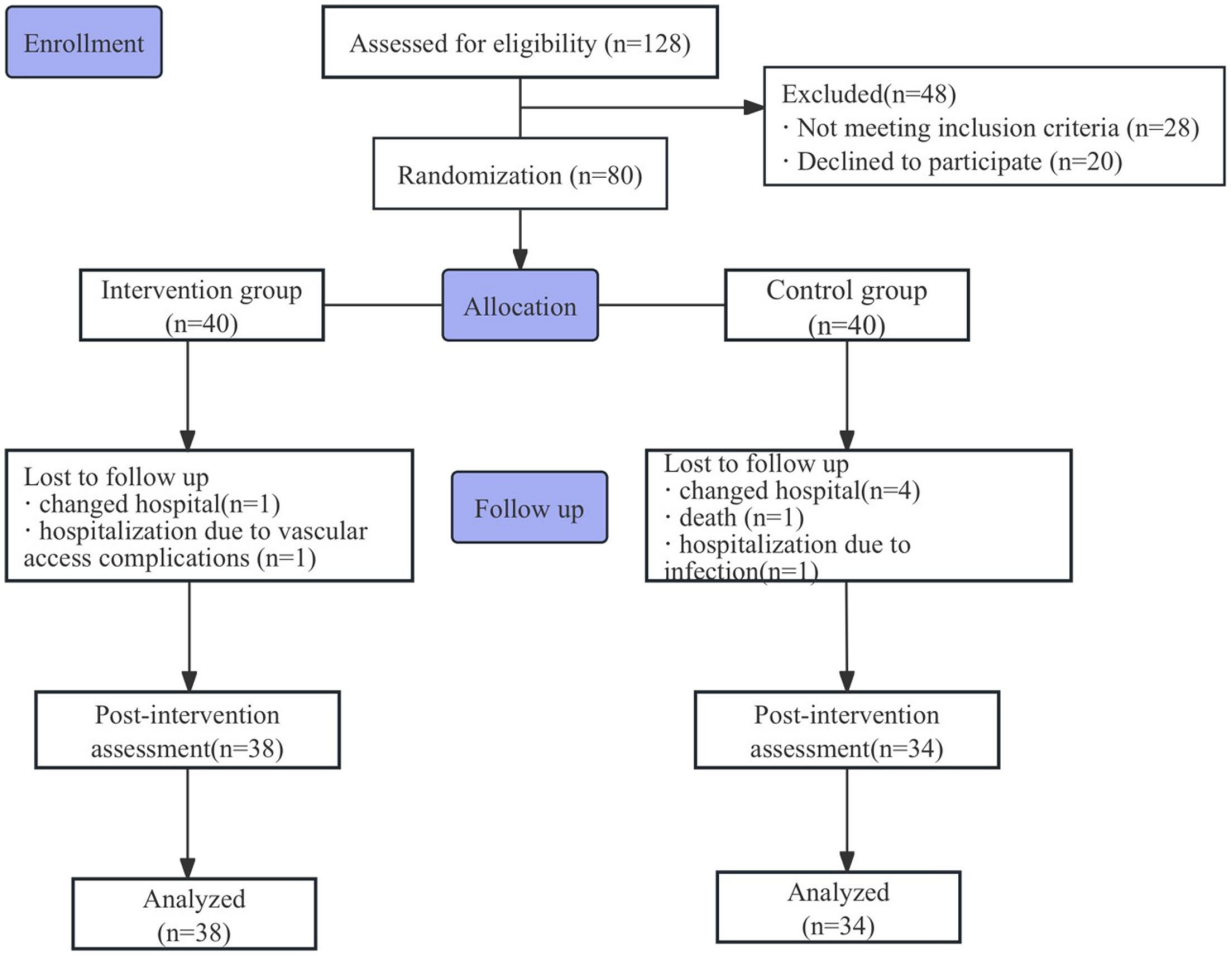
The flow of participants in the study.

A total of 128 MHD patients were assessed for eligibility at the First People’s Hospital of Lianyungang between November 2024 and February 2025. Following exclusions (*n* = 48), 80 participants were randomized. The participant flow is detailed in the CONSORT diagram (Figure 3).

#### 2.2.3 Sample size calculation

The sample size was estimated using the formula for comparing means between two independent samples: *N_1_* = *N_2_*= $2{\left[(\mu_{\alpha }+\mu_{\beta })/(\delta /\sigma )\right]}^{2}+{\mu_{\alpha }}^{2}/4$ , where *N_1_* and *N_2_* represent the required sample sizes for the two groups, $\delta =\mu_{1}-\mu_{2}$ denotes the difference between the two population means, and $\mu_{\alpha }$ and $\mu_{\beta }$ are the critical values corresponding to the significance level α and the Type II error probability β , respectively. Here, α is two-sided, while β is one-sided. Based on previously reported results (44), the parameters were set as δ=1.41−1.30=0.11 , and the sample standard deviation (s) was used to approximate the population standard deviation σ . With α=0.05 (two-sided) and β=0.10 , the corresponding critical values were $\mu_{\alpha }=1.96$ and $\mu_{\beta }=1.282$ . Accounting for an anticipated attrition rate of 10–20%, the final sample size was determined to be 80 participants (40 per group).

#### 2.2.4 Study design and randomization

This study employed a randomized controlled trial design. The randomization and allocation concealment procedures were as follows: an independent researcher generated a random sequence using SPSS 27.0 and prepared sequentially numbered, opaque, sealed envelopes for allocation concealment. Recruiting staff opened envelopes in sequential order to assign participants. Ultimately, 80 patients were equally allocated to the intervention and control groups. To prevent cross-contamination between groups, patient bed arrangements were appropriately adjusted during the study period after obtaining patient consent.

#### 2.2.5 Blinding

Due to the behavioral nature of the exercise adherence intervention, blinding of participants and intervention providers was not feasible. To minimize measurement bias and performance bias, all outcome assessors responsible for collecting primary and secondary outcome data remained blinded to group allocation. The researcher performing the final data analysis was also blinded to group assignment, and the dataset provided for analysis used labels “Group 1” and “Group 2” instead of actual group identities until the primary analysis was completed.

#### 2.2.6 Intervention methods

##### 2.2.6.1 Control group

Patients in the control group received standard MHD care per clinical guidelines. Dialysis nurses provided comprehensive health education encompassing: (1) blood pressure control techniques; (2) individualized fluid restrictions targeting interdialytic weight gain< 4% of dry weight; (3) personalized nutritional guidance. During sessions, nurses continuously monitored and documented vital signs (blood pressure, heart rate, oxygen saturation) and assessed vascular access status for infection and dysfunction. While healthcare staff educated patients and caregivers on exercise benefits and encouraged self-directed activities, no structured exercise regimens or monitoring devices were provided. All protocols adhered to hospital hemodialysis standards.

##### 2.2.6.2 Intervention group

The intervention group received the BCW theory-based exercise adherence intervention in addition to standard care. A multidisciplinary intervention team was established, consisting of eight members: one nephrologist, one exercise rehabilitation specialist, one cardiologist, three hemodialysis nurses (including one head nurse and two clinical nurses with over 5 years of experience), and 2 nursing graduate students. The intervention lasted for 12 weeks and was implemented as follows.

Prior to intervention initiation, the research team developed health education materials for MHD patients, which included printed manuals and instructional videos covering both home-based and intradialytic exercise protocols. Subsequently, exercise equipment such as recumbent cycles and sandbags were procured based on individual patient preferences and functional capacity. Through collaboration with biomedical engineers, the hospital’s existing mobile health platform, *Digital Therapeutics*, was adapted to address MHD patients’ characteristics and needs. The updated platform was evaluated through a testing phase involving five MHD patients, whose feedback was incorporated into final refinements. All intervention team members subsequently received training and competency assessment regarding the use of the enhanced platform.

On dialysis days, the protocol incorporated structured in-person group educational sessions. Key components covered proper timing and safety precautions to ensure patients correctly understood modalities and frequency. All participants received standardized training in Borg’s Rating of Perceived Exertion (RPE) scale for monitoring exercise intensity to optimize safety. The intervention protocol provided specialized intradialytic exercise equipment and real-time professional supervision to ensure exercise safety during hemodialysis.

On non-dialysis days, the *Digital Therapeutics* WeChat Mini Program delivered personalized health education and exercise diaries. The platform served dual purposes: sending exercise reminders and enabling comprehensive monitoring of exercise modalities, intensity, duration, and vital signs (pre−/intra−/post-activity), along with tracking any adverse events. Patients were guided to record body weight and other health metrics through the program. The platform’s knowledge base was enriched to allow patients to freely choose between text or audio formats. Exercise willingness was assessed regularly, and the frequency of educational content delivery could be manually adjusted via the backend based on patient engagement. Complementary WeChat group activities included: biweekly knowledge quizzes (administered via Wenjuanxing) with incentive rewards; peer interaction facilitation by encouraging exercise experience-sharing among patients; real-time troubleshooting guidance from healthcare providers and researchers to address home-exercise queries.

#### 2.2.7 Exercise prescription

##### 2.2.7.1 Intradialytic exercise

Aerobic exercise: Using a stationary cycle ergometer (MOTOmed viva2, Germany), intradialytic exercise was conducted three times per week with adjustable frequency based on patient tolerance. Sessions were performed 30 min to 2 h after dialysis initiation, with intensity progressively increased from low to moderate-high levels as measured by the Rating of Perceived Exertion (RPE) scale (12–14). Each session lasted 30–40 min.

Resistance training: Using sandbags, exercises included elbow flexion, elbow extension, wrist extension, ankle plantarflexion and dorsiflexion, supine straight leg raises, and side-lying leg lifts (see Appendix video for detailed demonstrations). Each exercise was performed for 10–15 repetitions per set, progressively increasing from an initial 1–2 sets to 3–5 sets.

##### 2.2.7.2 Non-dialysis day exercise

Based on pre-study assessments of patient preferences, baseline daily step counts, and collaboratively developed exercise prescriptions, patients self-selected exercise timing and modalities. Primary exercise forms included walking, cycling, and calisthenics, performed 2–3 times weekly for 30–60 min per session. The optimal timing was recommended as at least 2 h after meals, no less than 1 h before bedtime, or during morning/evening hours. Exercise intensity was maintained at 12–14 on the Rating of Perceived Exertion (RPE) scale.

#### 2.2.8 Ethical review

This study was approved by the Institutional Ethics Committee (approval no.: KY-20240409001-02) and registered at the Chinese Clinical Trial Registry (registration no.: ChiCTR2400091015). All participants provided written informed consent prior to enrollment.

### 2.3 Outcome measure

To provide a comprehensive assessment of the intervention’s effects, we implemented a multi-tiered outcome framework spanning four domains: Behavioral Targets, physical function, body composition, and laboratory biomarkers. This approach enables evaluation of the intervention’s impact, ranging from its immediate influence on target behaviors to its potential effects on physiological systems. The specific data collection methods, instruments, timepoints, and responsible personnel for all outcome measures are detailed in Table 1.

**Table 1:** Data collection methods, instruments, and timepoints for outcome measures.

Outcome measure	Instrument/Method	Assessor/Data source	Data collection timepoint				Specific protocol and details
			Week 0	Week 4	Week 8	Week 12	
Primary outcomes							
Exercise Adherence Score	Exercise adherence questionnaire	Researcher (Blinded)	**√**			**√**	Questionnaires were collected immediately upon completion and reviewed on-site for completeness.
Daily Average Step Count	The “WeRun” function within WeChat	Patient Self-report	**√**	**√**	**√**	**√**	The average daily step count over the 2 weeks preceding the intervention served as the baseline.
Secondary outcomes							
Physical function							
HGS	Dynamometer (Xiangshan Medical Equipment Co., Ltd., Guangdong, China)	Researcher (Blinded)	**√**			**√**	Before the scheduled dialysis sessions on Wednesdays or Thursdays.
6MWS	Digital stopwatch		**√**			**√**	
6MWD	6-Minute Walk Test (6MWT)		**√**			**√**	
Body composition							
SMI	Bioelectrical impedance analysis (BIA, InBody770; Biospace Co., Ltd., Seoul, Korea)	Researcher (Blinded)	**√**			**√**	At 30 min post-dialysis, following initial fluid redistribution.
Laboratory biomarkers							
Kt/V	Beckman Coulter DxA 5,000	Dialysis Nurse/Hospital Central Lab	**√**			**√**	Paired blood samples were drawn before and after the hemodialysis session to determine blood urea nitrogen (BUN) levels.
ALB			**√**			**√**	Pre-dialysis blood collection.
TyG			**√**			**√**	
CRP/NLR	Sysmex Mindray BC-7500		**√**			**√**	Pre-dialysis fasting blood samples (**≥**8 h fast).

All research staff involved in data collection received standardized training prior to the study commencement to ensure consistency and reliability.

#### 2.3.1 Primary outcome: behavioral targets

##### 2.3.1.1 Exercise adherence questionnaire

This study employed a questionnaire developed by Li (45), which comprises three dimensions: physical exercise adherence (6 items), exercise monitoring adherence (3 items), and proactive advice-seeking adherence (3 items), totalling 12 items. The scale has a scoring range of 12–48 points, with higher scores indicating stronger exercise adherence. The scale demonstrated excellent internal consistency with a global Cronbach’s *α* of 0.961, and the three dimensions had Cronbach’s *α* coefficients ranging from 0.834 to 0.952. The overall validity of the scale was 0.919. This questionnaire was selected based on its demonstrated reliability in MHD patients (27) (Cronbach’s α = 0.818) and validated factor structure in renal transplant recipients (46).

##### 2.3.1.2 Daily average step count

Patients carried their smartphones with them to record their total daily steps, including both dialysis and non-dialysis days. This objective metric was selected over subjective questionnaires for its ability to overcome recall bias, its clinical relevance as a predictor of patient outcomes, and its practicality as a simple, cost-effective measure of physical activity (47).

#### 2.3.2 Secondary outcomes: physical function

To assess whether improvements in exercise behavior translated into meaningful gains, we selected objective measures that are exercisable-responsive and prognostic in MHD patients to serve as direct evidence of the intervention’s success in improving overall functional status.

##### 2.3.2.1 Hand grip strength (HGS)

The standardized protocol required participants to assume an upright position with feet shoulder-width apart and upper limbs relaxed in anatomical neutral position (shoulder adduction ≤10°, elbow extension ≤5°). Participants performed maximum voluntary contractions sustained for 5 s using a dynamometer operated by the non-fistula extremity. Strict postural control prohibited extraneous body contact with the dynamometer during testing. Three trials with **≥**15-s rest intervals were conducted, recording the highest valid measurement. As a rapid and reliable measure of muscular strength and functional capacity, HGS is an independent predictor of critical clinical endpoints, including adverse outcomes and all-cause mortality, in the dialysis population (48, 49).

##### 2.3.2.2 6-Meter walking speed (6MWS)

Patients were timed while walking a 6-meter distance at their habitual walking pace. The test was repeated twice with adequate rest intervals, and the average time was calculated as the result. It was selected as it serves as an objective, integrative measure of lower extremity function and physiological reserve, while also being a well-validated predictor of mortality and cardiovascular events in MHD patients (50).

##### 2.3.2.3 6-Min walk test (6MWT)

The 6 MW was employed to evaluate functional capacity. Patients were instructed to walk repetitively along a 30-meter straight corridor at their maximal safe ambulatory speed (running expressly prohibited). Vital signs including heart rate, blood pressure, and oxygen saturation were monitored throughout the test. Research staff implemented real-time safety surveillance for clinical symptoms (dyspnea, angina, musculoskeletal pain) with predefined termination criteria: Patient-reported intolerable discomfort; Investigator-identified safety concerns. Personalized exercise prescriptions were developed according to individual patient characteristics based on the results, including total distance walked (in meters) and physiological parameter change 6MWT was selected as it provides a comprehensive assessment of cardiopulmonary function, systemic muscular endurance and strength, balance, it serves as a well-established measure of functional capacity for performing daily life activities (51).

#### 2.3.3 Secondary outcomes: body composition

##### 2.3.3.1 Skeletal Muscle Index (SMI)

It serves as a direct and standardized measure of muscle mass and constitutes a key component in the diagnostic criteria for sarcopenia (49). The patient’s SMI was assessed using bioelectrical impedance analysis (BIA, InBody770; Biospace Co., Ltd., Seoul, Korea). After completing dialysis treatment, patients were instructed to empty their bladder and bowels, have their height and weight measured, and remove any metal accessories or electronic devices that might interfere with the results. During the measurement, patients were instructed to remain still and avoid movement until the procedure was completed.

#### 2.3.4 Secondary outcomes: laboratory biomarkers

To complement the functional assessments, we also monitored routine laboratory biomarkers that are integral to the clinical management of MHD patients. These indicators were selected because they are objective, routinely measured, and may be favorably influenced by increased physical activity.

##### 2.3.4.1 Dialysis adequacy

Urea clearance index (Kt/V) was calculated using pre−/post-dialysis blood urea nitrogen (BUN) levels. Kt/V =−ln(R−0.008T)+(4−3.5R)×UF/W , where R represents the post−/pre-dialysis urea ratio, T is dialysis time (h), UF is ultrafiltration volume (L), and W is the patient’s post-dialysis weight (kg) (52). Kt/V was included as an outcome measure given its prognostic value and its documented responsiveness to exercise interventions (53).

##### 2.3.4.2 Microinflammatory status

Assessed via C-reactive protein (CRP) and neutrophil-to-lymphocyte ratio (NLR). Patients with MHD often experience chronic inflammation, which contributes to various complications (54). We assessed both CRP, a classic inflammatory biomarker, and the NLR, a more recent metric (55), to provide a comprehensive evaluation of this microinflammatory state.

##### 2.3.4.3 Nutritional status

Determined by serum albumin (ALB) levels. ALB was included as an outcome measure due to its dual role in assessing nutritional status and inflammatory burden (56).

##### 2.3.4.4 Glucose-lipid metabolism

Assessed using the triglyceride-glucose index ( $\mathrm{TyG}=\ln[\begin{array}{l} \;\text{fasting triglycerides}\;(\mathrm{mg}/\mathrm{dL})\\ \times \text{fasting glucose}\;(\mathrm{mg}/\mathrm{dL})∕2 \end{array}]$ ). We included the TyG index as an objective measure to assess the metabolic benefits of exercise. Its established role as a marker of insulin resistance and CVD risk (57) allows us to evaluate whether improved exercise adherence translates into improved metabolic health.

### 2.4 Statistical methods

All statistical analyses were performed using SPSS 27.0 (IBM Corp., Armonk, NY, USA), with a two-tailed significance level of *α* = 0.05. Normally distributed continuous variables were analyzed using samples *t*-tests and reported as mean ± standard deviation (SD). Nonparametric comparisons employed Mann–Whitney *U* tests with results expressed as median (interquartile range [IQR]). Categorical variables were summarized as frequencies (percentage).

For the longitudinal analysis of daily average step counts (measured at baseline, week 4, 8, and 12), a Generalized Estimating Equations (GEE) model was fitted to assess the main effects of group and time, as well as their interaction. Post-hoc pairwise comparisons with Bonferroni adjustment were conducted to interpret any significant interaction effects.

## 3 Results

### 3.1 Phase I: outcomes of the intervention development process

#### 3.1.1 Semi-structured interviews

Thirteen patients were interviewed. The interview data were analyzed using the COM-B model as the framework, leading to the identification of three main themes and twelve sub-themes: (1) Capability factors (Deficient knowledge of exercise rehabilitation; Inadequate self-management competencies; Negative perception of physical fitness status). (2) Opportunity factors (Demand for individualized exercise prescriptions; Environmental constraints on physical activity; Availability of social support systems; Experiences of social detachment). (3) Motivation factors (Presence of kinesiophobia; Recognition of exercise’s therapeutic value; Established exercise habits and prior experiences; Anticipated health benefits from participation; Enhanced perception of patient role in self-care).

Following research team analysis and discussion, seven core intervention functions from the BCW framework were selected for adoption: Education, Persuasion, Incentivization, Training, Environmental Restructuring, Modeling, and Enablement. Correspondingly, 23 specific BCTs were formulated, establishing the intervention framework (see Supplementary Table S2).

#### 3.1.2 Summary of best evidence

Ten publications were included: one clinical decision document (58), two guidelines (10, 59), three expert consensus statements (9, 60, 61), two systematic reviews (22, 62), and two randomized controlled trials (24, 63) (see Supplementary Tables S3–S6 for the quality assessments). Twenty-four best practices were summarized across six domains: establishing a multidisciplinary team, assessing patient willingness and barriers, creating personalized exercise plans, providing socio-environmental support, implementing monitoring and feedback, and applying mobile health technology.

Evidence levels and recommendation grades were evaluated using the JBI system. The preliminary intervention framework was then refined based on this evidence to form the initial protocol draft.

#### 3.1.3 Expert panel meeting

Fourteen experts participated. Panel demographics were as follows: all held intermediate-to-senior titles; 50% (*n* = 7) held PhDs, 43% (*n* = 6) held master’s degrees, and 7% (*n* = 1) held a bachelor’s degree. The expert authority coefficient (Cr) attained 0.91, indicating high expertise credibility, while the Kendall’s Coefficient of Concordance (W = 0.383, *p* < 0.01) reflecting a good degree of consensus and active engagement among the experts (Supplementary Table S7).

The expert panel reached the following collective conclusions: (1) refine textual expression by eliminating redundant content; (2) specify concrete operational measures in the intervention protocol to enhance clinical applicability; (3) specify the types of mobile health (mHealth) technologies used during the intervention process (e.g., smartphones, tablets, or other electronic devices) to ensure technical feasibility and patient adaptability; (4) shift from fixed to flexible intervention scheduling to accommodate patients’ individualized needs and time availability. Specific Recommendations with Corresponding Revisions are presented in Supplementary Table S8. Through research team deliberations and expert panel discussions, the final version of the exercise adherence intervention protocol for MHD patients has been established, comprising 3 first-level indicators, 12 s-level indicators, and 29 third-level indicators (Table 2).

**Table 2:** Finalized exercise adherence intervention program for MHD patients.

Intervention domain	Intervention functions	Objectives	Implementation strategies	Temporal framework
1. Capacity building	Education training implementation	1.1 Exercise knowledge dissemination and cognitive misconception rectification.	1.1.1 Instructional manual distribution and *Digital Therapeutics* WeChat Mini Program navigation guidance. Bedside health education on exercise adherence benefits and sedentary risk mitigation.	Week 1; Patient-initiated sessions.
			1.1.2 Education on intradialytic and non-dialysis day exercise modalities, safety considerations, and guideline-recommended exercise prescriptions.	
		1.2 Physical capacity evaluation and collaborative goal setting.	1.2.1 Regular exercise assessments are conducted for patients, including: Medical evaluation encompassing (Disease status: vital signs monitoring, primary renal pathology identification, dialysis vintage, and assessment of comorbidities; Clinical Examination Results: recent biochemical parameters, cardiopulmonary function evaluation outcomes; Treatment Parameters: medication regimens, prescribed dialysis parameters, vascular access surveillance). Baseline physical activity level: habitual movement patterns, physical activity profiles.	Pre-exercise initiation, clinical status changes, treatment adjustments; and at weeks 4, 8, 12 of intervention.
			1.2.2 The research team collaboratively developed individualized exercise plans with each patient based on: age, cultural background, personal preferences, resource accessibility, Baseline physical activity levels (mean daily step count during 2-week pre-intervention period).	
		1.3 Stage-matched behavioral intervention and habit formation.	1.3.1 Behavioral change stage assessment: Stage-matched intervention (precontemplation/contemplation: motivational interviewing + health literacy enhancement; preparation/action: positive reinforcement + evidence-based information scaffolding).	At intervention weeks 1, 4, 8, 12.
			1.3.2 Utilizing the *Digital Therapeutics* WeChat mini program to deliver activity log prompts; Instructing patients/caregivers in non-dialysis day recordings (activity type/duration/perceived exertion/intensity); Activating WeChat pedometer-based step tracking for self-monitoring competency building.	Throughout the entire intervention period.
		1.4 Motor skill instruction and injury prevention protocol.	1.4.1 Educating patients/caregivers in heart rate monitoring and Borg CR-10 scale application during non-dialysis days; Implementing graded intensity progression protocol (initial low-intensity phase with transition to moderate-intensity target: RPE 12–14).	Throughout the entire intervention period.
			1.4.2 Delivering structured exercise skill education: intradialytic exercis (recumbent cycling/resistance training with sandbags and bands); Non-Dialysis Day Activities (cycling/stair-climbing/jogging/square dancing). The exercise protocol includes pre-exercise warm-up and post-exercise cool-down activities to prevent sports injuries and alleviate pain.	
			1.4.3 Periodic evaluation of physical functional capacity: Adjustment of exercise plans based on patient needs and capabilities.	
2. Opportunity creation	Education Training Modeling Implementation	2.1 Exercise environment adaptation and behavioral modification.	2.1.1 Patient-consented bed assignment optimization: Strategic relocation of exercise-motivated patients to enhance peer interaction and mutual encouragement dynamics.	
			2.1.2 Intradialytic exercise infrastructure provision: Supply of therapeutic equipment (resistance bands/sandbags/recumbent cycles) during HD sessions; Nurse-supervised implementation with real-time monitoring logs (exercise duration/intensity/pre-post BP-glucose/completion rate/adverse events/vascular access integrity).	
			2.1.3 Digital health support system: Multidisciplinary WeChat group (patients/caregivers/nephrologists/exercise therapists) delivering exercise tutorials and video demonstrations; Off-dialysis remote coaching through video feedback and structured Q&A chains.	
		2.2 Exercise skill reinforcement and behavioral consolidation.	2.2.1 WeChat-based knowledge surveillance: Implementation of Wenjuanxing surveys to assess exercise-related knowledge mastery; Targeted educational reinforcement for identified gaps; Conducting bedside knowledge evaluations during dialysis for technologically challenged patients.	Weekly during weeks 1–4.
			2.2.2 Digital education resource deployment: Video delivery via the *Digital Therapeutics* WeChat Mini Program and WeChat groups demonstrating intradialytic/non-dialysis day exercise modalities, facilitate anytime access to reinforce exercise skills.	Throughout the entire intervention period.
		2.3 Social support mobilization and exercise confidence enhancement.	2.3.1 Enhance exercise education for the patient’s primary caregiver, correct their misconceptions, and encourage them to accompany and supervise the patient’s exercise completion on non-dialysis days.	Throughout the entire intervention period.
			2.3.2 Provide verbal praise when the patient achieves exercise goals; if goals are unmet, assist the patient in analyzing reasons and promptly address barriers.	
			2.3.3 Group patients by residential area/community to establish exercise groups for peer-supported workouts; encourage/assist them in joining local walking groups, square dance teams, etc., via social media.	
			2.3.4 Encourage high-performing patients to share exercise tips via WeChat groups, and motivate less adherent patients to engage in physical activity.	At intervention weeks 4, 8, 12.
3. Motivation enhancement	Education Persuasion Incentivization Modeling Environmental Restructuring	3.1 Dynamic supervision and feedback for behavioral maintenance.	3.1.1 On non-dialysis days, verify the timely submission of patients’ exercise logs. For cases of non-compliance, initiate telephone follow-ups to provide reminders and ascertain the underlying reasons for non-adherence.	Throughout the entire intervention period.
			3.1.2 Generate trend graphs to visualize patients’ exercise completion rates. Collect and plot longitudinal data on physical function indicators (including grip strength and 6-meter walking speed), skeletal muscle mass, as well as biochemical markers(C-reactive protein, albumin) and dialysis adequacy at baseline and week 12. Provide patients with feedback regarding their positive outcomes.	
			3.1.3 Implement operant conditioning protocol with baseline weekly 10-point allocations: apply daily non-compliance penalties (−1) for missed exercise logging; award journaling compliance incentives (+1); activate gamified leaderboard system with 4-week cycles culminating in redeemable exercise-related incentives (top 5) and verbal reinforcement for sustained adherence.	
		3.2 Positive emotion guidance and negative experience avoidance.	3.2.1 Monitor patients’ post-exercise conditions and subjective feedback, and adjust subsequent exercise plans accordingly to mitigate kinesiophobia.	Throughout the entire intervention period.
			3.2.2 Guide patients to recognize positive health changes following exercise participation; Encourage experience sharing and peer interaction through WeChat support groups.	
		3.3 Exercise role model establishment and self-identity development.	3.3.1 Inform patients that their active participation in exercise will establish them as role models within their group, thereby motivating fellow patients to engage in physical activity	At intervention weeks 4, 8, 12.
			3.3.2 For patients with low adherence, demonstrate the improvement metrics and exercise completion rates of high-adherence peers within the same group. Additionally, arrange psychological counseling to help them overcome emotional barriers while providing targeted encouragement to achieve their goals.	
		3.4 Exercise belief reinforcement and self-efficacy enhancement.	3.4.1 Organize monthly expert lectures on disease management and exercise science; Conduct interactive quiz sessions with practical demonstrations by high-performing patients, awarding exercise gear for active participation.	6 sessions, 30–60 min each, every 2 weeks.
			3.4.2 Regularly assess patients’ current behavioral stages, review goal achievement and identify barriers from the previous phase, assist in problem-solving, and establish phased short-term/long-term goals to support incremental target realization.	At intervention weeks 4, 8, 12.
		3.5 Behavioral goal review and maintenance promotion.	3.5.1 Provide patients with visual feedback using line charts to demonstrate exercise goal completion rates and achieved outcomes, thereby reinforcing confidence in sustained exercise adherence.	
			3.5.2 Develop success case profiles of patients achieving significant exercise adherence improvements and publicly display through WeChat patient communities and ward multimedia terminals to motivate behavioral modifications in non-compliant populations.	

### 3.2 Phase II: outcomes of the intervention application and evaluation

#### 3.2.1 Participant flow and baseline characteristics

All 72 enrolled participants completed the study. No statistically significant differences were observed in baseline characteristics between the groups (*p* > 0.05). Details are presented in Table 3.

**Table 3:** Comparison of baseline characteristics between intervention and control groups.

Characteristics	Intervention group	Control group	Statistical measures	*p*-value
Age (years)	51.95 ± 10.86	55.41 ± 12.30	−1.269^1^	0.212
Gender				
Male	22 (57.9%)	15 (44.1%)	1.363^2^	0.243
Female	16 (42.1%)	19 (55.9%)		
Work status				
Full-time employment	9 (23.7%)	8 (23.5%)	<0.001^2^	0.988
Retirement	29 (76.3%)	26 (76.5%)		
Average monthly income (CNY)				
<3,000	11 (28.9%)	14 (41.2%)	1.184^2^	0.277
≥3,000	27 (71.1%)	20 (58.8%)		
Marital status				
Married	32 (84.2%)	30 (88.2%)	0.023^2^	0.879
Unmarried/Other	6 (15.8%)	4 (11.8%)		
Education level				
Primary school and below	10 (26.3%)	11 (32.4%)	1.254^3^	0.534
Middle school	12 (31.6%)	13 (38.2%)		
High school and or beyond	16 (42.1%)	10 (29.4%)		
Medical insurance type				
Urban employee/Resident	31 (81.6%)	24 (70.6%)	1.202^2^	0.273
Rural cooperative or other	7 (18.4%)	10 (29.4%)		
Dialysis vintage (years), [*M* (*P*25, *P*75)]	5.5 (2.75, 7)	6 (3, 8)	−0.40^3^	0.968
Primary disease				
Glomerulonephritis	13 (34.2%)	6 (17.9%)	3.213^2^	0.373
Diabetes mellitus	5 (13.2%)	7 (20.6%)		
Hypertension	17 (44.7%)	16 (47.1%)		
Other	3 (7.9%)	5 (14.7%)		
Comorbidities (types)				
<2	15 (39.5%)	13 (38.2%)	0.012^2^	0.914
≥2	23 (60.5%)	21 (61.8%)		
Exercise habits				
Daily average step count	2,398 (1,634, 4,169)	2014 (1,324, 4,087)	−0.936^3^	0.349

^1^*t*-value; ^2^*χ*^2^ value; ^3^Z-value.

#### 3.2.2 Effects on behavioral targets

At baseline, there were no significant differences between the intervention and control groups in either exercise adherence scores (median [IQR]: 18 [16, 20] vs. 18 [14.8, 24], *p* = 0.405) or daily average step counts (median [IQR]: 2398 [1,634, 4,169] vs. 2014 [1,324, 4,087], *p* = 0.349). After 12 weeks of intervention, the exercise adherence score was significantly higher in the intervention group (35.3 ± 4.33) compared to the control group (22.6 ± 3.62), with a statistically significant difference (*p* < 0.001).

To assess the longitudinal intervention effects, a Generalized Estimating Equations (GEE) model was applied to the daily average step count data. The model revealed a significant main effect of time (Wald *χ*^2^ = 125.229, *p* < 0.001), indicating substantial changes in steps across all participants over the study period. The main effect of group was not statistically significant (Wald *χ*^2^ = 6.050, *p* = 0.430). Most critically, a significant group-by-time interaction was found (Wald *χ*^2^ = 60.804, *p* < 0.001), confirming that the trajectory of change differed significantly between the two groups.

The parameter estimates from the GEE model delineated a clear pattern of this interaction. The negative and significant interaction coefficients for the control group across all follow-ups (Week 4: *β* = −0.088, *p* = 0.003; Week 8: *β* = −0.291, *p* < 0.001; Week 12: *β* = −0.329, *p* < 0.001) indicate that the increase in step counts over time was significantly less pronounced in the control group compared to the intervention group. Within the intervention group, the significant positive time coefficients (all *p* < 0.001) demonstrate a strong and sustained increase from baseline.

Between-group comparisons further solidified the intervention’s effect. The intervention group’s step counts were significantly higher than the control group’s at all post-baseline follow-up assessments, as evidenced by the significant interaction terms. Furthermore, the increasing magnitude of these interaction coefficients (from −0.088 to −0.329) indicates that the between-group advantage was sustained over the duration of the intervention.

In summary, the GEE analysis confirms that the intervention was effective in producing a superior and sustained increase in daily physical activity compared to the control group. Details are presented in Table 4.

**Table 4:** Comprehensive analysis of daily step counts: descriptive statistics and model comparisons.

Time point	Intervention group	Control group	Comparison type	Model estimates (95% CI)	*p*-value
Baseline (W0)	2,398 (1,634, 4,169)	2014 (1,324, 4,087)	–	–	–
Week 4 (W4)	3,062 (2052, 4,757)	2,199 (1,671, 4,546)	Cross-sectional	MD: 1501.27 (831.38, 2171.16)	<0.001
			GEE model	β: −0.088(−0.147, −0.030)	0.003
			W0–W4	MD: 1412.79 (1226.28, 1599.30)	<0.001
Week 8 (W8)	3,439 (2,704, 5,105)	1944 (1,609, 4,116)	Cross-sectional	MD: 1651.68 (997.07, 2306.29)	<0.001
			GEE Model	β: −0.291 (−0.372, −0.209)	<0.001
			W0–W8	MD: 1030.50 (851.30, 1209.70)	<0.001
Week 12 (W12)	4,021 (3,073, 5,247)	2092 (1,672, 4,226)	Cross-sectional	MD: 1690.09 (1018.40, 2361.79)	<0.001
			GEE model	β: −0.329 (−0.418, −0.239)	<0.001
			W0–W12	MD: 545.95 (418.88, 673.02)	<0.001

Data are presented as Median (interquartile range, IQR). Cross-sectional: Mean difference (MD) in step counts between groups at each time point. GEE model: β coefficient from the Group × Time interaction term, representing the difference in change (log-scale) from baseline between the Control and Intervention (reference) groups; a negative β indicates lesser increase in the Control group. W0–W4/W8/W12: MD in step count change from baseline between groups over the specified period. All models used an unstructured correlation matrix.

#### 3.2.3 Effects on physical function and body composition

At baseline, no significant differences were observed between the groups in HGS, 6MWS, 6MWT distance and SMI (all *p* > 0.05). After the 12-week intervention, the intervention group showed significantly greater improvements than the control group in HGS (*p* < 0.05), 6MWS (*p* < 0.001), 6MWT (*p* < 0.001), and SMI (*p* < 0.05). Complete results are presented in Table 5.

**Table 5:** Between-group comparisons of secondary outcomes.

Item	Time point	Intervention group	Control group	*P*-value
Physical function				
HGS (kg)	Pre-intervention	28.30 (21.28, 36.15)	23.65 (20.03, 30.03)	0.129
	Post-intervention	30.60 (25.35, 41.28)	23.55 (20.70, 30.98)	0.004
6MWS (m/s)	Pre-intervention	1.08 (0.95, 1.19)	0.98 (0.91, 1.10)	0.155
	Post-intervention	1.22 (1.14, 1.28)	1.03 (0.90, 1.16)	<0.001
6MWT (m)	Pre-intervention	393.26 ± 55.41	376.94 ± 54.28	0.212
	Post-intervention	441 (403.75, 455.25)	368.50 (316.75, 414.25)	<0.001
Body composition				
SMI (kg/m^2^)	Pre-intervention	7 (6.4, 8.1)	6.7 (6.0, 7.5)	0.103
	Post-intervention	7.21 ± 0.91	6.72 ± 0.97	0.019
Laboratory biomarker				
Kt/V	Pre-intervention	1.34 ± 0.21	1.37 ± 0.24	0.590
	Post-intervention	1.44 (1.31, 1.53)	1.31 (1.18, 1.52)	0.034
CRP (mg/L)	Pre-intervention	2.94 (1.24, 4.54)	2.85 (1.21, 5.71)	0.756
	Post-intervention	1.68 (0.92, 3.23)	2.63 (0.96, 7.08)	0.121
NLR	Pre-intervention	3.14 (2.19, 3.55)	3.21 (2.49, 4.76)	0.234
	Post-intervention	2.94 (2.12, 3.84)	3.27 (2.68, 3.93)	0.245
Alb(g/L)	Pre-intervention	40.55 ± 3.10	39.37 ± 3.14	0.115
	Post-intervention	41.64 ± 2.81	39.70 ± 3.35	0.009
TyG index	Pre-intervention	9.41 ± 1.07	9.74 ± 1.12	0.195
	Post-intervention	8.74 ± 0.62	9.21 ± 0.71	0.004

Kt/V: Target: ≥1.4 (Indicates adequate dialysis); CRP: Per lab standard: <3.0 mg/L; Alb(g/L): Normal range: 35–50 g/L; NLR: The commonly used clinical cut-off was applied: <3.0; TyG: The established cut-off for insulin resistance was applied: ≥8.5.

#### 3.2.4 Effects on laboratory biomarkers

No significant baseline differences were observed in any laboratory measures between the groups (all *p* > 0.05).

After the 12-week intervention, the intervention group showed significantly higher Kt/V values than the control group (*p* = 0.034). No significant between-group differences were found in CRP or NLR levels post-intervention (*p* > 0.05). For nutritional and metabolic markers, the intervention group demonstrated significantly higher albumin levels and lower TyG index values compared to the control group (both *p* < 0.05). Detailed results are presented in Table 5.

## 4 Discussion

### 4.1 A BCW-based intervention: scientific foundation and clinical feasibility

Promoting sustained exercise adherence in MHD patients necessitates interventions that address the complex behavioral determinants underlying physical activity. To meet this challenge, we developed a 12-week intervention guided by the BCW framework, which provided a systematic methodology for translating identified barriers into targeted strategies across capability, opportunity, and motivation domains. The protocol was co-designed with a multidisciplinary team to ensure clinical relevance and safety from its inception.

The resulting intervention employs a hybrid delivery model that integrates initial in-person guidance with ongoing remote supervision. This design was specifically crafted to provide continuous patient support while accommodating real-world staffing constraints. Through structured expert panel review, the protocol was further refined to confirm its methodological rigor and practical applicability within routine clinical practice.

### 4.2 Sustained exercise adherence: evidence for behavioral maintenance mechanisms

Our results confirm the efficacy of the BCW model in improving exercise adherence. The intervention group demonstrated significant increases in both adherence scores and objectively measured daily step counts.

Our findings reveal a distinctive cumulative adherence pattern that differs meaningfully from previous exercise interventions in MHD patients. While structured interventions have consistently demonstrated an ability to initiate behavior change—as evidenced by Liu’s motivation-oriented ADOPT model (27) and Malhotra’s goal-setting approach (24)—these approaches share a common limitation: intervention effects typically peak within 8–12 weeks before beginning to decline (64, 65). In contrast, our BCW-based intervention generated progressively strengthening effects throughout the entire 12-week study period (Wald *χ*^2^ = 60.804, *p* < 0.001), demonstrating maintained improvement where other approaches have faltered.

This sustained benefit appears to stem from our intervention’s theoretical grounding in the BCW framework. The distinction becomes particularly evident when comparing technology-mediated approaches. Anand (65) treated mobile health technology as a simple adjunct to conventional exercise prescription and found no significant benefits. In our intervention, however, the digital platform functioned as an integrated component of the BCW system—not merely as a monitoring tool but as a mechanism for enabling self-regulation through digital self-monitoring and sustaining social support via regular clinician feedback.

The significant group-by-time interaction provides robust evidence for this dynamic treatment effect. The intervention established a positive feedback loop through consistent behavior activation, self-efficacy enhancement, and reinforced social support—mechanisms that collectively bridged the intention-behavior gap that often undermines long-term adherence in conventional exercise programs. The progressive nature of improvement suggests that the 12-week intervention period may represent a crucial window for establishing autonomous exercise habits. Future studies should investigate whether these gains persist beyond the active intervention phase, which would provide further evidence for true habit formation.

### 4.3 From adherence to physiological adaptation: functional and body composition improvements

Our 12-week intervention produced significant functional improvements with important implications. Patients in the intervention group demonstrated statistically significant improvement in HGS compared to both baseline and control group levels (*p* < 0.05), consistent with previous studies investigating progressive resistance training in MHD populations (66, 67). This enhancement in muscular strength may translate to better performance in daily activities and reduced fall risk associated with sarcopenia (48).

The intervention group also showed substantial gains in both 6MWT performance and 6MWS The improvement in gait speed carries particular prognostic significance, as epidemiological evidence indicates that each 0.1 m/s increase is associated with a 25.7% reduction in all-cause mortality risk (50). Together with HGS and 6MWT distance—both established independent predictors of adverse outcomes in MHD patients, these coordinated functional improvements demonstrate that our intervention successfully translated behavioral changes into meaningful physiological adaptations.

These functional benefits were complemented by positive changes in body composition. The intervention group achieved a final SMI of 7.21 ± 0.91 kg/m^2^, with the prevalence of low SMI decreasing from 21.1 to 15.8%. This improvement is clinically relevant given that low SMI values affect 20–55% of MHD patients and are associated with increased mortality risk (68). The reduction in low SMI prevalence suggests that our BCW-based approach may effectively counteract the progression of sarcopenia in this vulnerable population.

The concordant improvements across multiple domains—muscular strength, mobility, endurance, and body composition—provide compelling evidence that our behavior change intervention produced comprehensive physiological benefits with potential implications for long-term clinical outcomes in MHD patients.

### 4.4 From behavior to biology: laboratory correlates of improved clinical status

Our intervention generated significant improvements across key laboratory parameters, demonstrating the physiological impact of enhanced exercise adherence. The intervention group showed a statistically significant improvement in Kt/V compared to controls, indicating enhanced dialysis efficacy. This improvement likely originates from multiple mechanisms: first, regular exercise promotes peripheral circulation and increases muscle tissue perfusion, potentially improving clearance efficiency of uremic toxins during dialysis (44); second, the observed increase in SMI indicates enhanced muscle mass, and as muscle serves as a critical metabolic organ, this preservation may thereby optimize systemic metabolic regulation (69).

In contrast to some previous studies reporting the beneficial effects of exercise on inflammatory markers (CRP, NLR) levels, our study found no statistically significant differences between groups. It suggests that in the MHD population, the physiological benefits derived from improved exercise adherence may preferentially impact metabolic and functional pathways, whereas the more entrenched chronic inflammatory state—driven by factors such as the uremic milieu and dialysis-related bioincompatibility—may require interventions of greater duration or intensity than the 12-week behavioral program implemented here (30, 70, 71). It is noteworthy that persistently elevated levels of these inflammatory markers have been significantly associated with adverse cardiovascular outcomes (72, 73) and have emerged as a major contributor to morbidity and mortality in this population. This insight directs future research toward exploring combined intervention strategies, potentially integrating exercise with targeted anti-inflammatory approaches, to effectively modulate inflammation in this population.

Serum albumin levels significantly increased in the intervention group, confirming the positive impact of exercise on nutritional status in MHD patients (66). This improvement likely stems from multiple mechanisms: regular exercise promotes protein anabolism while reducing muscle catabolism, enhances gastrointestinal motility to improve nutrient absorption, and may stimulate appetite to increase dietary intake. Additionally, the structured nutritional guidance delivered through the Digital Therapeutics platform may have synergistically contributed to these biomarker improvements through combined intervention strategies.

Most notably, the TyG index demonstrated significantly greater improvement in the intervention group. As a validated surrogate marker of insulin resistance and predictor of cardiovascular outcomes (57, 74), the reduction in TyG index indicates amelioration of insulin resistance, which may directly mitigate associated metabolic risk factors such as hyperglycemia and dyslipidemia that are strongly linked to adverse cardiovascular outcomes. Our findings align with evidence from type 2 diabetes populations where exercise-induced reduction in TyG index drives improvements in glucolipid metabolism (75, 76), while extending these observations to the MHD population. The decreased TyG index provides a plausible pathophysiological explanation for exercise’s cardioprotective effects and, given its accessibility and low cost, suggests its potential utility as a practical surrogate endpoint for evaluating metabolic benefits of exercise interventions in clinical practice.

These coordinated improvements in both nutritional status and metabolic profile underscore the multifaceted physiological benefits achievable through enhanced exercise adherence. The BCW-based intervention not only promoted behavioral change but also translated these changes into concrete physiological advantages, addressing two critical pathological pathways in MHD patients through a unified behavioral approach.

### 4.5 Limitations

This study has several limitations. First, while daily step counts provided an objective measure of physical activity volume, the absence of intensity monitoring through heart rate or accelerometer-based metabolic equivalents prevents a more comprehensive assessment of exercise dose–response relationships. Second, the single-center design, though ensuring protocol consistency and implementation fidelity, may limit the generalizability of our results to other clinical settings with different patient demographics or healthcare resources. Future studies should incorporate wearable devices for comprehensive activity monitoring, implement multi-center designs with extended follow-up periods, and employ component-analysis approaches to identify the most effective intervention elements.

Third, the 12-week duration prevented assessment of long-term adherence patterns beyond the intervention period. Finally, as an effectiveness trial evaluating a bundled intervention, we did not analyze specific causal pathways between exercise adherence and physiological improvements.

Future studies should incorporate multi-center designs with extended follow-up periods and utilize advanced monitoring to capture both activity volume and intensity.

## 5 Conclusion

This study demonstrates that a BCW theory-based exercise adherence intervention provides an effective approach for managing MHD patients. The intervention not only improved exercise behavior but also translated these gains into meaningful functional and physiological benefits, including enhanced physical capacity, improved dialysis adequacy, and better metabolic profile. These findings highlight the value of using a systematic behavioral framework to address exercise promotion challenges in this complex population. Future research should verify these results in multi-center settings and investigate the long-term sustainability of these benefits.

## Data Availability

The raw data supporting the conclusions of this article will be made available by the authors, without undue reservation.
