# Supplementary material for: Effectiveness of a BCW theory-based exercise adherence intervention protocol in Chinese maintenance hemodialysis patients: a randomized controlled trial
Source: Front Public Health. 2025 Dec 12;13:1700057. doi: 10.3389/fpubh.2025.1700057 (PMC12741123; doi:10.3389/fpubh.2025.1700057)
Supplement: Supplementary file 1 [file Table_1.DOCX]

Supplementary Material

# Supplementary Tables

Table S1 The PIPOST Framework for Scoping the Evidence Synthesis

| Component | Definition | Application in This Study |
| --- | --- | --- |
| P: Population | The target patient group for the evidence-based intervention. | Adult patients on maintenance hemodialysis (MHD). |
| I: Intervention | The evidence-based action or approach being implemented. | Strategies and methods aimed at improving exercise adherence. |
| P: Professionals | The individuals responsible for applying the evidence in practice. | Clinical healthcare providers (e.g., nephrologists, nurses, rehabilitation therapists). |
| O: Outcomes | The measures used to evaluate the effectiveness of the implementation. | Primary: Exercise adherence. Secondary: Program completion rate, participation level, dropout rate. |
| S: Setting | The context or environment where the evidence is applied. | Hospitals (e.g., dialysis centers) and home-based settings. |
| T: Type of Evidence | The forms and levels of evidence considered for implementation. | Clinical decision support, practice guidelines, evidence summaries, systematic reviews, expert consensuses, and randomized controlled trials. |

| COM-B | Semi-structured Interviews | Intervention Function | BCTs |
| --- | --- | --- | --- |
| Capability | 1.Deficient knowledge of exercise rehabilitation.  2.Negative perception of physical fitness status.  3.Inadequate self-management competencies. | Education  Training  Enablement | 4.1 Instruction on how to perform the behavior.  5.1 Information about health consequences.  9.3 Comparative imagining of future outcomes.  1.1 Goal setting (behavior)  1.4 Action planning.  1.6 Discrepancy between current behavior and goal.  2.3 Self-monitoring of behavior.  4.1 Instruction on how to perform the behavior. |
| Opportunity | 1.Demand for individualized exercise prescriptions.  2.Environmental constraints on physical activity.  3.Availability of social support systems.  4.Experiences of social detachment. | Environmental Restructuring  Training  Enablement  Modelling | 1.2 Problem solving  7.1 Prompts/cues  12.1 Restructuring the physical environment.  3.2 Social support (practical)  3.3 Social support (emotional)  5.4 Monitoring of emotional consequences  6.1 Demonstration of the behavior  11.2 Reduce negative emotions |
| Motivation | 1.Presence of kinesiophobia.  2.Enhanced perception of patient role in self-care.  3.Recognition of exercise's therapeutic value.  4.Anticipated health benefits from participation.  5.Established exercise habits and prior experiences; | Education  Persuasion  Enablement  Persuasion  Incentivisatio  Training  Environmental Restructuring  Modelling | 1. Goal setting (behavior)  1.2 Problem solving  1.3 Goal setting (outcome)  2.2 Feedback on behavior  2.7 Feedback on outcome(s) of behavior  5.1 Information about health consequences  5.2 Salience of consequences  9.3 Comparative imagining of future outcomes.  10.2 Reward (behavior)  2.3 Self-monitoring of behavior  6.2 Social comparison  8.3 Habit formation  9.1 Credible source |

Table S2 Intervention Framework Developed from Semi-structured Interview Findings

Table S3 Quality Appraisal Results of Clinical Guidelines

| Included Literature | Standardized percentage of each dimension (%) | | | | | | No. of Domains ≥60% | No. of Domains ≥30% | Recommendation Grade |
| --- | --- | --- | --- | --- | --- | --- | --- | --- | --- |
|  | Scope and Purpose | Stakeholder Involvement | Rigor of Development | Clarity of Presentation | Applicability | Editorial Independence |  |  |  |
| Baker | 97.2 | 86.1 | 84.4 | 75 | 85.4 | 62.5 | 6 | 6 | A |
| KDIGO | 83.3 | 94.4 | 86.5 | 80.6 | 87.55 | 90 | 6 | 6 | A |

Table S4 Quality Assessment of Systematic Reviews

| Evaluation Item | Included Literature | |
| --- | --- | --- |
|  | Li | Zhang |
| 1. Was the evidence-based question clearly and explicitly stated? | Yes | Yes |
| 2. Were the inclusion criteria appropriate for the evidence-based question? | Yes | Yes |
| 3. Was the search strategy appropriate? | Yes | Yes |
| 4. Were the databases or resources for literature search adequate? | Yes | Yes |
| 5. Were the criteria for quality assessment of literature appropriate? | Yes | Yes |
| 6. Was quality assessment performed independently by two or more reviewers? | Yes | Yes |
| 7. Were measures taken to minimize errors in data extraction? | Yes | Yes |
| 8. Were the methods for study synthesis appropriate? | Yes | Yes |
| 9. Was the potential for publication bias assessed? | No | Yes |
| 10. Were the policy or practice recommendations based on the systematic review results? | Yes | Yes |
| 11. Were the proposed directions for future research appropriate? | Yes | Yes |

Table S5 Quality Appraisal of Expert Consensus Documents

| Evaluation Item | Included Literature | | |
| --- | --- | --- | --- |
|  | Chinese Association of Rehabilitation Medicine | The Committee of Renal Rehabilitation, Society of Rehabilitation Physicians, Chinese Medical Doctor Association | Bennett |
| 1.Is the source of viewpoints/statements clearly indicated? | Yes | Yes | Yes |
| 2.Do the viewpoints originate from influential experts in the field? | Yes | Yes | Yes |
| 3. Are the proposed perspectives centered on the interests of the relevant study population? | Yes | Yes | Yes |
| 4. Are the conclusions based on analytical results, and is the presentation of viewpoints logical? | Yes | Yes | Yes |
| 5. Is reference made to existing literature? | Yes | Yes | Yes |
| 6. Are there any inconsistencies between the proposed viewpoints and previous literature? | Yes | Yes | Yes |

Table S6 Methodological Quality Assessment of Included Randomized Controlled Trials

| Evaluation Item | Evaluation Results | |
| --- | --- | --- |
|  | Greenwood | Malhotra |
| 1. Was true random allocation used for the samples? | Yes | Yes |
| 2. Was allocation concealment implemented? | Yes | Yes |
| 3. Were the groups comparable at baseline? | Yes | Yes |
| 4. Were the subjects blinded？ | N/A | N/A |
| 5. Were the intervention providers blinded? | N/A | N/A |
| 6. Were the outcome assessors blinded? | Yes | Yes |
| 7. Apart from the intervention being verified, were the adopted measures identical across groups? | Yes | Yes |
| 8. Was follow-up complete? If not, were strategies used to address incomplete follow-up? | Yes | Yes |
| 9. Were all randomly assigned samples included in the conclusion analysis? | Yes | Yes |
| 10. Were consistent methods used to assess the outcome measures for each group？ | Yes | Yes |
| 11. Were the outcome measurement tools reliable？ | Yes | Yes |
| 12. Were the data analysis methods appropriate? | Yes | Yes |
| 13. Was the study design appropriate? Was the study conduct and data analysis performed rigorously？ | Yes | Yes |

Table S7 Expert Participation, Authority Coefficient, and Coordination Coefficient

| Item | Expert Authority Coefficient | Expert Enthusiasm | | Expert Coordination Coefficient | | |
| --- | --- | --- | --- | --- | --- | --- |
|  |  | Questionnaire Recovery Rate (%) | Questionnaire Effective Rate (%) | Kendall’ W | Chi-square Test Value | *P*-value |
| Expert Meeting | 0.91 | 100% | 93.3% | 0.383 | 256.61 | <0.01 |

Table S8 Expert Comments and Revisions

| Identified Limitations in the Current Program | Proposed Improvement |
| --- | --- |
| 1.Personalized exercise prescriptions should be developed through multidisciplinary collaboration. | 1.The prescribing clinicians for exercise interventions have been expanded from the original "exercise physiologists and researchers" to an interdisciplinary clinical team comprising nephrologists, cardiologists, exercise rehabilitation specialists, and clinical researchers. |
| 2.The assessment of baseline physical activity levels should employ quantitative measures. | 2.The WeChat Sport fitness tracking feature was employed to document patients' daily step counts during a 2-week pre-intervention monitoring phase, with the mean value serving as the baseline physical activity levels. |
| 3.The mobile health (mHealth) modalities were not specified. | 3.Incorporated the application of the *Digital Therapeutics* WeChat Mini Program. |
| 4.The established exercise assessment protocol should be supplemented with comprehensive cardiopulmonary function metrics, including functional capacity evaluation (6-minute walk test), myocardial contractility assessment (left ventricular ejection fraction [LVEF] via echocardiography), and New York Heart Association (NYHA) functional classification. Additionally, advanced cardiac diagnostics comprising coronary computed tomography angiography (CTA) and 24-hour ambulatory electrocardiographic monitoring (Holter) must be incorporated. | 4.Following research team deliberations, the 6-minute walk test (6MWT) was selected as the primary assessment metric considering resource availability constraints. |
| 5.Health education should not be confined to exercise-related knowledge but rather evolve into multidimensional health literacy frameworks. | 5.Augment educational content related to patient fluid management, dietary interventions, and other relevant clinical care domains. |
| 6.Could the tri-daily transmission of WeChat-based exercise adherence reminders potentially induce message fatigue and intervention intolerance among patients? | 6.Remove this entry and add an additional reminder for patients with uncompleted daily tasks. |
| 7.Is the application of Wenjuanxing-based digital assessments methodologically appropriate for patients with limited literacy levels? | 7.Paper-based questionnaires were added, with bedside paper surveys or researcher-assisted completion for patients experiencing difficulties using Wenjuanxing (an online survey platform)." |
| 8."The division of exercise zones” is not clinically feasible. | 8.After discussion, the protocol was amended to: “Adjust bed assignments for willing patients upon consent”. |
| 9.Will a penalty point system for patients who fail to achieve targets negatively impact their motivation? | 9.The system was modified to a reward-based point scheme, eliminating punitive measures. |

**Table S9 CONSORT 2010 Checklist: Customized for Your RCT**

| **Section/Topic** | **Item No** | **Checklist item** | **Location in**  **Manuscript** |
| --- | --- | --- | --- |
| **Title and abstract** | 1a | **☑**Identification as a randomised trial in the title | Title page |
|  | 1b | **☑**Structured summary of trial design, methods, results, and conclusions (for specific guidance see CONSORT for abstracts) | Lines 16-44 |
| **Introduction** |  |  |  |
| Background and objectives | 2a | **☑**Scientific background and explanation of rationale | Lines 92-107 |
|  | 2b | **☑**Specific objectives or hypotheses | Lines 108-115 |
| **Methods** |  |  |  |
| Trial design | 3a | **□**Description of trial design (such as parallel, factorial) including allocation ratio | N/A |
|  | 3b | **□**Important changes to methods after trial commencement (such as eligibility criteria), with reasons | N/A |
| Participants | 4a | **☑**Eligibility criteria for participants | Lines 280 |
|  | 4b | **☑**Settings and locations where the data were collected | Lines 306 |
| Interventions | 5 | **☑**The interventions for each group with sufficient details to allow replication, including how and when they were actually administered | Lines 340 |
| Outcomes | 6a | **☑**Completely defined pre-specified primary and secondary outcome measures, including how and when they were assessed | Lines 416 |
|  | 6b | **□**Any changes to trial outcomes after the trial commenced, with reasons | N/A |
| Sample size | 7a | **☑**How sample size was determined | Lines 314 |
|  | 7b | **□**When applicable, explanation of any interim analyses and stopping guidelines | N/A |
| Randomisation |  |  |  |
| Sequence generation | 8a | **☑**Method used to generate the random allocation sequence | Lines 314 |
|  | 8b | **□**Type of randomisation; details of any restriction (such as blocking and block size) | N/A |
| Allocation concealment mechanism | 9 | **☑**Mechanism used to implement the random allocation sequence (such as sequentially numbered containers), describing any steps taken to conceal the sequence until interventions were assigned | Lines 314 |
| Implementation | 10 | **☑**Who generated the random allocation sequence, who enrolled participants, and who assigned participants to interventions | Lines 314 |
| Blinding | 11a | **☑**If done, who was blinded after assignment to interventions (for example, participants, care providers, those assessing outcomes) and how | Lines 333 |
|  | 11b | **☑**If relevant, description of the similarity of interventions | Lines 341 |
| Statistical methods | 12a | **☑**Statistical methods used to compare groups for primary and secondary outcomes | Lines 535 |
|  | 12b | **□**Methods for additional analyses, such as subgroup analyses and adjusted analyses | N/A |
| **Results** |  |  |  |
| Participant flow (a diagram is strongly recommended) | 13a | **☑**For each group, the numbers of participants who were randomly assigned, received intended treatment, and were analysed for the primary outcome | **Figure 3** |
|  | 13b | **☑**For each group, losses and exclusions after randomisation, together with reasons | **Figure 3** |
| Recruitment | 14a | **☑**Dates defining the periods of recruitment and follow-up | Lines 306 |
|  | 14b | **□**Why the trial ended or was stopped | N/A |
| Baseline data | 15 | **☑**A table showing baseline demographic and clinical characteristics for each group | **Table 3** |
| Numbers analysed | 16 | **□**For each group, number of participants (denominator) included in each analysis and whether the analysis was by original assigned groups | N/A |
| Outcomes and estimation | 17a | **□**For each primary and secondary outcome, results for each group, and the estimated effect size and its precision (such as 95% confidence interval) | N/A |
|  | 17b | **□**For binary outcomes, presentation of both absolute and relative effect sizes is recommended | N/A |
| Ancillary analyses | 18 | **□**Results of any other analyses performed, including subgroup analyses and adjusted analyses, distinguishing pre-specified from exploratory | N/A |
| Harms | 19 | **☑**All important harms or unintended effects in each group (for specific guidance see CONSORT for harms) | N/A |
| **Discussion** |  |  |  |
| Limitations | 20 | **☑**Trial limitations, addressing sources of potential bias, imprecision, and, if relevant, multiplicity of analyses | Lines 879 |
| Generalisability | 21 | **☑**Generalisability (external validity, applicability) of the trial findings | Lines 879 |
| Interpretation | 22 | **☑**Interpretation consistent with results, balancing benefits and harms, and considering other relevant evidence | Lines 678 |
| **Other information** |  |  |  |
| Registration | 23 | **☑**Registration number and name of trial registry | Lines 411 |
| Protocol | 24 | **☑**Where the full trial protocol can be accessed, if available | **Table 2** |
| Funding | 25 | **☑**Sources of funding and other support (such as supply of drugs), role of funders | Lines 926 |

**
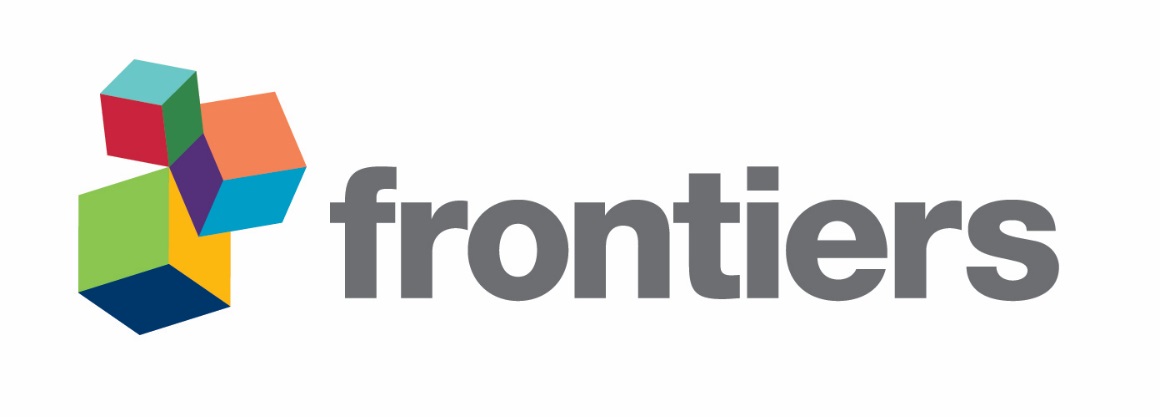
**
